# MRI identification of anatomical distribution of periosteal entrapment and its clinical outcomes in pediatric distal tibial Salter-Harris type II fractures

**DOI:** 10.3389/fspor.2025.1738452

**Published:** 2026-01-05

**Authors:** Hengheng Zhang, Xiaoming Wang, Ming Yang, Xing Tong, Jinwang Dong, Bing Wang

**Affiliations:** 1 Department of Pediatric Orthopedics, Xi'an Jiaotong University, Xi'an, Shaanxi, China; 2 School of Medicine, Yan'an University, Yan'an, Shaanxi, China

**Keywords:** distal tibia, growth disturbance, MRI, periosteal entrapment, physeal fractures

## Abstract

**Objective:**

This study aimed to characterize the spatial distribution and imaging features of periosteal entrapment in pediatric distal tibial Salter-Harris (S-H) type II fractures using magnetic resonance imaging (MRI) and to evaluate its clinical outcomes.

**Methods:**

This retrospective study analyzed 20 cases of distal tibial S-H type II fractures between 2015 and 2024. All patients underwent post-injury MRI examinations (including T1-weighted, T2-weighted, and proton density-weighted imaging sequences). The precise location of periosteal entrapment was recorded and mapped to the four quadrants of the epiphysis. All patients were divided into three groups according to the treatment method: the conservative treatment group, the closed reduction and percutaneous fixation (CR-PF) group, and the open reduction and internal fixation (ORIF) group. Outcomes evaluated included fracture healing, growth disturbances (including bone bridge formation, angular deformity, and limb-length discrepancy), and the American Orthopaedic Foot and Ankle Society (AOFAS) score at the final follow-up (≥6 months).

**Results:**

A total of 20 patients (16 boys and 4 girls) with an average age of 10.65 ± 2.41 years (range: 2–13 years) were included in this study, and the mean follow-up period was 22.53 months. Post-injury MRI showed periosteal entrapment in all patients, with the anterolateral quadrant being the main entrapment site (15/20, 75%). During the follow-up period, 2 patients developed growth disturbances: one patient who underwent ORIF showed a bone bridge formation on imaging, while the other patient who received CR-PF presented with ankle varus deformity without evidence of bone bridge formation. At the final follow-up, the assessment of AOFAS scores revealed no significant difference in functional outcomes among the three groups (*P* = 0.951).

**Conclusion:**

Distal tibial S-H type II physeal fractures are at high risk of concomitant periosteal entrapment, which is localized predominantly to the anterolateral corner of the distal tibial physis. Conservative management, CR-PF, and ORIF resulted in comparable functional outcomes without significantly increasing the risk of growth disturbances, indicating that residual entrapped periosteum may not adversely affect fracture healing or long-term prognosis.

## Introduction

Distal tibial physeal fractures are one of the most common lower extremity injuries in children, with Salter-Harris (S-H) type II fractures the most prevalent. This fracture pattern carries a high risk of complications, including premature physeal closure (PPC), progressive angular deformity, and limb-length discrepancy (1, 2). Traditionally, a residual physeal gap >3 mm after closed reduction has been considered an indicator of potential periosteal entrapment and as a threshold for advocating open reduction and internal fixation (ORIF) to mitigate the risk of growth arrest (3, 4). In cases of suspected periosteal entrapment, MRI is recommended as the superior imaging modality due to its ability to clearly demonstrate the entrapped structures within the physeal gap on multiplanar T2-weighted sequences (5). However, there is still controversy about whether the release of the entraped periosteum in open surgery actually improves the prognosis of fractures (6). Despite the significant clinical implications of periosteal entrapment, consensus remains lacking regarding its precise anatomical distribution at the physis and its definite impact on long-term prognosis. Therefore, the purposes of this study were: (1) to delineate the anatomic distribution pattern of periosteal entrapment in distal tibial S-H type II fractures using MRI, and (2) to compare the clinical outcomes of three different treatment methods—conservative treatment, closed reduction with percutaneous fixation (CR-PF), and ORIF—through American Orthopaedic Foot and Ankle Society (AOFAS) score.

## Patients and methods

### Patients

This retrospective cohort study reviewed 33 cases of distal tibial S-H type II physeal fractures treated between 2015 and 2024. Among these, 20 patients with distal tibial S-H type II fractures combined with periosteal entrapment, identified via 3.0 T MRI performed within 72 h post-injury, were included as the study subjects. The inclusion criteria were as follows: (1) Acute distal tibial S-H II physeal injury; (2) Periosteal entrapment confirmed by MRI; (3) Follow-up duration >6 months with complete follow-up data. Exclusion criteria: (1) Other S-H types (types I, III, or IV); (2) MRI not performed or MRI showing absence of periosteal entrapment; (3) Other fractures around the ankle joint on the same side (e.g., tarsal fractures, foot fractures); (4) Follow-up duration <6 months or incomplete clinical data.

### Magnetic resonance imaging

**Figure 1:**
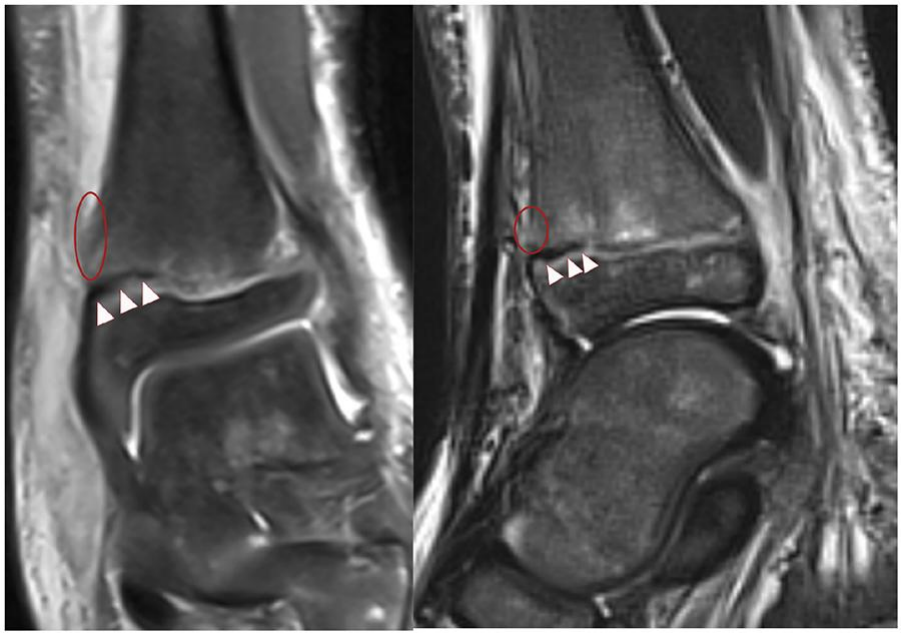
MRI showed periosteal entrapment in the anteromedial quadrant of the distal tibial physis (white arrow), along with a periosteal defect in the metaphysis (red circle).

- All affected ankle joints were imaged using a 3.0-Tesla scanner. The imaging protocol included the following sequences: Sagittal T2-weighted fat-suppressed sequence [repetition time (TR)/echo time (TE) = 4,390/92 ms], Sagittal T1-weighted sequence (TR/TE = 500/9.5 ms), coronal proton-weighted fat-suppressed sequences (TR/TE = 2,000/30 ms), Axial T2-weighted sequence (TR/TE = 4,000/89 ms). Scan parameters were set as follows: scanning layer thickness 2.5–5 mm, layer spacing 3–5, Image Size 320-320–512-512. To evaluate the location of periosteal entrapment, the distal tibial epiphysis was conceptually divided into four quadrants (anteromedial, anterolateral, posteromedial, and posterolateral). The quadrant containing the entrapped periosteal (including any metaphyseal periosteal defect) was recorded (Figure 1).

### Investigated variables

Patient demographic and clinical data, including gender, age, affected side, imaging findings, and treatment modality, were collected through retrospective review of medical records. Imaging evaluation was based on radiographs and MRI. Two experienced pediatric orthopedic surgeons independently reviewed all MRI scans and serial radiographs to assess the presence and precise location of periosteal entrapment. Any discrepant interpretations were resolved through discussion to reach a consensus.

Closed reduction was initially attempted in all patients. Acceptable reduction was defined as the restoration of normal anatomical alignment (angulation < 5°) on anteroposterior and lateral ankle radiographs. If an acceptable closed reduction was achieved and maintained, conservative treatment with cast or brace immobilization was administered. Unstable fractures were managed with CR-PF. If significant residual physeal gap persisted after closed reduction, accompanied by unacceptable ankle joint alignment, conversion to ORIF was performed.

Growth disturbance was defined as the occurrence of any of the following during the follow-up period: (1) confirmed physeal bar formation on radiography, CT, or MRI; (2) progressive angular deformity of the ankle (>5° compared to the contralateral side); (3) limb length discrepancy >1 cm. Growth disturbances were diagnosed by pediatric orthopedic surgeons based on imaging findings or the need for reoperation to correct functional impairment of the affected limb. All patients were followed for a minimum of 6 months. Functional outcomes of the affected limb were assessed at the final follow-up using the AOFAS score, which is graded as excellent (90–100), good (80–89), fair (70–79), or poor (<70) (7).

### Statistical methods

Statistical analyses were performed using SPSS software (version 21.0). The normality of the data was assessed using tests such as the Shapiro–Wilk test, with results expressed as mean ± standard deviation (M ± SD) or median and interquartile range (IQR). For non-normally distributed AOFAS scores, between-group comparisons were conducted using the Kruskal–Wallis *H* test. The significance level was set at *α* = 0.05.

## Results

The study cohort consisted of 20 patients (16 boys and 4 girls) with a mean age of 10.65 ± 2.41 years (range: 2–13 years). The mean follow-up duration was 22.53 months. Nine fractures involved the left side and eleven the right. Treatment modalities consisted of conservative management in 8 patients, CR-PF in 5 patients, and ORIF in 7 patients. Concomitant fibular fractures were present in 7 cases (see Table 1 for details).

**Table 1:** Patient baseline characteristics and outcomes.

Patient	Age(years)	Sex(Female, F/Male, M)	Injured side(Right, R/Left, L)	Concomitant fibular fracture(Yes/No)	Location of entrapped periosteum(MRI)	Treatment methods	Follow-up time(months)	Growth disturbance(Yes/No)	AOFAS score
1	10.81	F	R	Yes	AM	Conservative treatment	44.47	No	100
2	12.74	M	L	No	AL	Conservative treatment	27.50	No	100
3	12.39	M	R	No	AL	CR-PF	20.97	Yes	100
4	13.03	M	R	No	AL	Conservative treatment	13.13	No	98
5	10.19	M	R	No	AL	Conservative treatment	16.10	No	100
6	2.96	M	L	No	AM	CR-PF	6.27	No	90
7	10.99	M	L	No	AL	Conservative treatment	16.67	No	89
8	10.08	M	L	Yes	AM	ORIF	18.43	Yes	90
9	8.63	M	L	No	AL	Conservative treatment	15.23	No	99
10	11.34	M	R	No	AL	CR-PF	6.04	No	98
11	9.66	F	R	No	AL	Conservative treatment	6.03	No	100
12	9.01	F	R	Yes	AM	Conservative treatment	6.73	No	100
13	8.96	M	R	Yes	AM	ORIF	89.47	No	100
14	12.65	M	R	Yes	AL	ORIF	50.50	No	100
15	10.25	F	R	No	AL	ORIF	46.47	No	94
16	8.78	M	L	Yes	AL	ORIF	25.70	No	100
17	13.08	M	R	Yes	AL	CR-PF	9.87	No	100
18	12.65	M	L	No	AL	ORIF	10.25	No	100
19	13.56	M	L	No	AL	ORIF	7.28	No	100
20	11.27	M	L	No	AL	CR-PF	13.51	No	100

MRI, magnetic resonance imaging; AM, anteromedial; AL, anterolateral; CR-PF, closed reduction and percutaneous fixation; ORIF, open reduction and internal fixation.

Periosteal entrapment was confirmed by MRI in all 20 cases of distal tibial S-H type II physeal fractures. The entrapment was located in the anterolateral quadrant of the distal tibial epiphysis in 15 cases (75%), and in the anteromedial quadrant in 5 cases (25%). Radiographic evidence of fracture healing was observed in all 20 patients within approximately 6–8 weeks. Two patients developed growth disturbances of the distal tibia. One patient, despite undergoing ORIF, exhibited radiographic evidence of a physeal bar formation and subsequently required revision surgery due to progressive ankle angular deformity. The other patient with a growth disturbance presented with a varus deformity of the ankle, but no physeal bar was detected on imaging.

Clinical outcomes were assessed using the AOFAS scoring scale, with 19 excellent and 1 good. Comparison of functional outcomes among the conservative treatment group (*n* = 8), CR-PF group (*n* = 5), and ORIF group (*n* = 7) showed no significant difference in AOFAS scores (*P* = 0.951) (Table 2). No instances of infection, neurovascular injury, joint stiffness, compartment syndrome, or iatrogenic physeal injury were observed in any of the patients.

**Table 2:** Comparison of clinical outcomes among three patient groups: AOFAS scores.

Treatment methods	Total(*n* = 20)	Conservative treatment(*n* = 8)	CR–PF(*n* = 5)	ORIF(*n* = 7)	*P*
AOFAS score	100.00 (98.00, 100.00)	100.00 (98.25, 100.00)	100.00 (94.00, 100.00)	100.00 (94.00, 100.00)	0.951

AOFAS, American Orthopaedic Foot and Ankle Society; CR-PF, closed reduction and percutaneous fixation; ORIF, open reduction and internal fixation.

## Discussion

Distal tibial physeal fractures are among the most common lower extremity injuries in children. If not managed appropriately, they can lead to serious growth disturbances such as PPC, resulting in limb length discrepancy or angular deformity of the ankle (8–10). Previous studies have identified multiple factors predictive of poor prognosis, among which periosteal entrapment is considered a key factor (11–14). Using MRI, this study further clarified the predilection for anterolateral periosteal entrapment in patients with distal tibial S-H type II fractures. The findings of this study can be summarized as follows: (1) a high incidence of periosteal entrapment was observed in distal tibial S-H type II fractures, with a predominant anterolateral location; (2) all three treatment modalities achieved favorable and comparable functional outcomes (*P* = 0.951), while radiographic physeal bars were observed only in the ORIF group.

Conventionally, radiographs are sufficient for the initial evaluation of most physeal injuries; however, MRI is required for definitive diagnosis when assessing the presence of periosteal entrapment within the fracture gap (5). Nevertheless, due to the high cost of MRI, it is not routinely used as an imaging modality in the acute injury setting. In this study, all 20 cases underwent post-injury MRI examinations. Among them, 15 cases showed entrapped periosteum located in the anterolateral quadrant, while 5 cases exhibited entrapment in the anteromedial quadrant. This distribution pattern may be related to the biomechanical characteristics of distal tibial physeal displacement caused by the supination injury mechanism. In this injury pattern, the periosteum attached to the tension side (typically anterolateral) of the metaphysis is stripped from the fracture fragment by external force. During reduction attempts, the avulsed periosteum becomes entrapped within the physeal gap (15, 16). Simultaneously, compressive stress on the epiphyseal side manifests as a localized S-H type V axial compression injury, which damages the germinal layer cells in the central or anteromedial region of the physis known as Kump's bump, thereby leading to PPC. This mechanism suggests that there may not be a direct causal relationship between periosteal entrapment and PPC.

**Figure 2:**
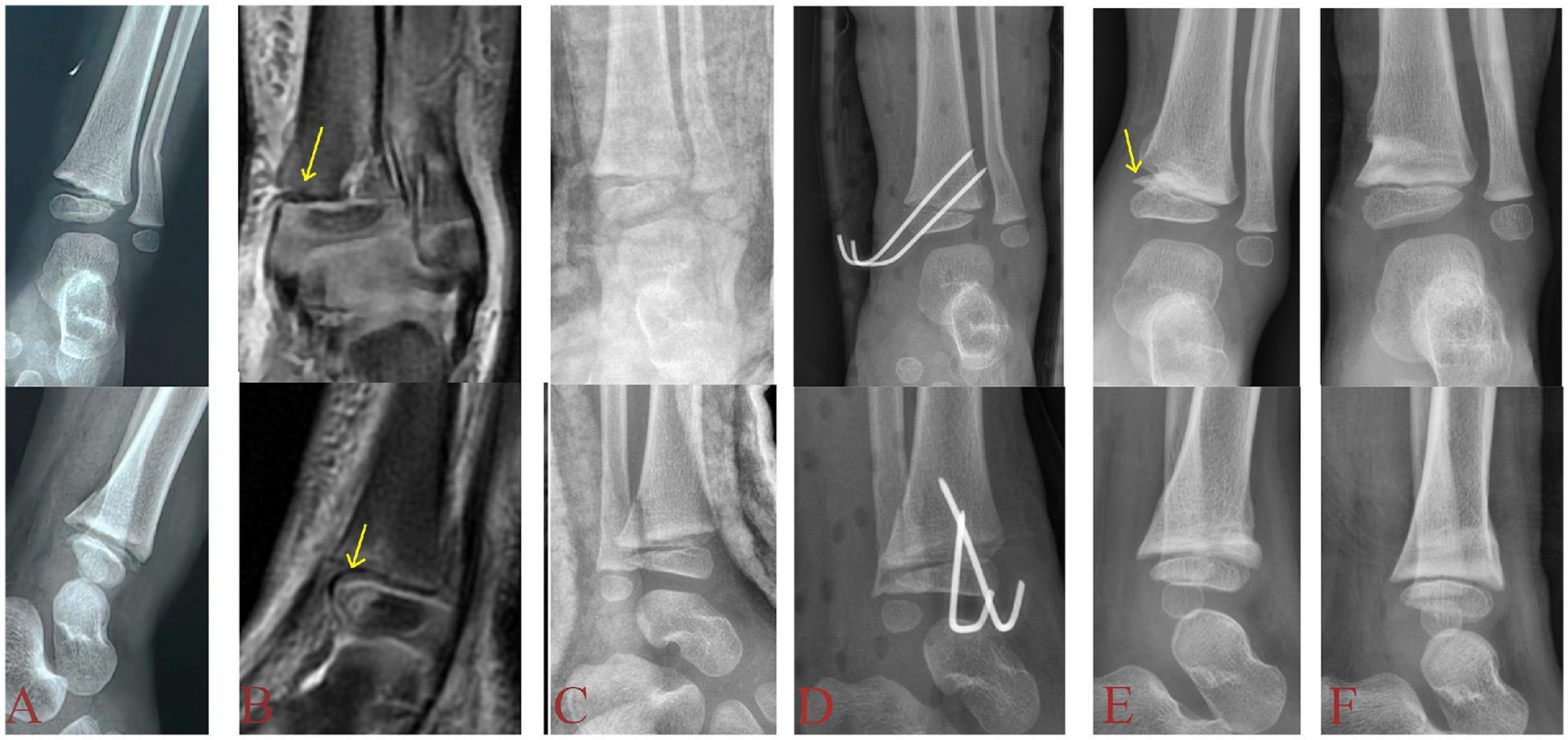
A 2-year-old boy presented with an open distal tibial S-H type II fracture (PEER injury). **(A)**: Initial radiograph shows widening of the anteromedial physeal gap of the distal tibia. **(B)**: MRI reveals periosteal entrapment within the anteromedial quadrant of the distal tibial physis (yellow arrow). **(C)**: Following closed reduction, repeat radiograph shows persistent widening of the anteromedial physeal gap. **(D)**: CR-PF was performed under general anesthesia, achieving satisfactory postoperative joint alignment. **(E)**: Two-month follow-up radiograph shows the “periosteal entrapment sign”—a horizontally oriented bony protrusion with density comparable to normal bone in the anteromedial distal tibia, distinct from the circumferential callus formation seen in subperiosteal ossification (yellow arrow). **(F)**: Six-month follow-up demonstrates excellent ankle function without angular deformity.

Because periosteal entrapment has been regarded as a critical risk factor for poor outcome in distal tibial S-H II fractures, it has traditionally served as an indication for ORIF, in order to lower the risk of subsequent complications (8, 17, 18). However, the causal relationship between periosteal entrapment and PPC may not be absolute. In this study, despite all patients who underwent ORIF achieving anatomical reduction, one case still developed PPC. Furthermore, comparative analysis of AOFAS functional scores among the three groups showed comparable functional outcomes (*P* = 0.951). This finding suggests that periosteal entrapment may not be the direct cause of PPC: only one patient showed radiographically visible bone bridge formation during follow-up, and this case occurred in the ORIF group. Moreover, we observed that the bone bridge formation was not located at the initial site of periosteal entrapment, but rather at the Kump's bump area of the distal tibial epiphysis. These findings indicate that the entrapped periosteum itself is not invariably the fundamental cause of growth disturbance, and that other mechanisms, such as external force causes damage to the epiphyseal germ layer cells, may play a more decisive role. The other case complicated by angular deformity without bone bridge formation. Studies (19) suggest that the underlying cause may be attributed to overgrowth on the affected side. This phenomenon likely results from either increased blood supply during healing or stimulated growth due to weight-bearing at the fracture site, ultimately leading to angular deformity of the ankle joint. However, the authors considered the cause may be related to asymmetric activity of the distal tibial physis or an imbalance in growth between the distal tibial and fibular physes. Although entrapped periosteum may act as a mechanical barrier, this study demonstrates that it is not always sufficient to disrupt the critical germinal layer of the physis for bone bridge formation. Furthermore, in some patients who did not undergo ORIF, follow-up radiographs revealed a characteristic “periosteal entrapment sign”—namely, a horizontally oriented bony protrusion within the physeal gap with density similar to normal bone, distinct from the circumferential callus seen in subperiosteal ossification (Figure 2). This appears to confirm the findings of Gruber et al. (20): the entrapped periosteum, recognized as a foreign body, becomes displaced by the continuing growth of the physis during the healing process, thereby forming the distinctive “periosteal entrapment sign” observed radiographically. This phenomenon is particularly evident in children with substantial growth potential. In summary, the role of periosteal entrapment in the long-term prognosis of distal tibial physeal fractures requires further investigation. Given that ORIF may carry risks of surgical site infection and increased healthcare costs, CR-PF should be prioritized for such patients when satisfactory joint alignment can be achieved and maintained. If satisfactory joint alignment can be achieved, anatomic reduction of the physis with mandatory removal of the entrapped periosteum is not mandatory—especially in younger children with substantial growth potential.

Based on the findings of this study, patients with MRI-confirmed periosteal entrapment who were managed with non-ORIF approaches (conservative treatment or closed reduction with internal fixation) did not demonstrate inferior outcomes compared to those who underwent ORIF. Therefore, treatment selection for pediatric distal tibial S-H type II fractures with periosteal entrapment requires careful consideration. The mere presence of periosteal entrapment should not be viewed as an absolute indication for ORIF. The primary indication for ORIF remains the inability to achieve or maintain acceptable closed reduction—specifically, when satisfactory articular alignment cannot be restored through conservative means. If closed reduction achieves satisfactory ankle alignment and the fragment is stable, conservative treatment or CR-PF appears to be the more reasonable option—even in the presence of MRI-confirmed periosteal entrapment. This approach not only reduces the risk of complications associated with open surgery and decreases healthcare costs, but also aligns with our case series findings demonstrating comparable clinical functional outcomes across the three treatment modalities (*P* = 0.951) without increasing the risk of growth disturbance.

This study has several limitations. First, This retrospective study employed a non-randomized treatment allocation strategy, implementing a stepwise approach based on fracture reduction quality and ankle alignment. Such a non-randomized design inherently carries potential selection bias, which should be considered when interpreting subsequent research findings. Despite this limitation, the outcome data from our cohort suggest that the current view of periosteal impaction as the direct and primary cause of postfracture growth impairment warrants reevaluation. Future prospective randomized controlled trials will facilitate comparative analysis of clinical outcomes across different treatment modalities. Second, the sample size of 20 cases is insufficient to accurately describe the anatomical distribution features of periosteal entrapment and the generalizability of the findings. Future, studies with larger sample sizes are required to validate the current conclusions and should include subgroup analyses based on the location of periosteal entrapment (e.g., anterolateral vs. anteromedial) to explore potential regional differences in growth outcomes. Additionally, the cellular-level evolution of entrapped periosteum and the local molecular mechanisms within the entrapment site warrant further in-depth investigation. Third, the follow-up duration was relatively short, with some patients not being monitored until skeletal maturity. However, it is noteworthy that bone bridges typically become detectable through radiographic examination approximately six months after the initial injury. Fourth, in patients treated with CR-PF, the absence of routine postoperative MRI evaluation makes it uncertain whether the entrapped periosteum was released, potentially biasing the results. However, based on intraoperative observations, the periosteum entrapped within the fracture gap typically exhibits a curled or folded configuration rather than a flattened morphology, making it mechanically difficult to dislodge through manual reduction alone. Therefore, in this study, the status of the periosteum in such cases was still classified as “unreleased” postoperatively. It should be noted that remaining growth potential is a key determinant of growth disturbance, yet this study did not analyze the variable of age. We propose that periosteal entrapment, as a common concomitant phenomenon in distal tibial physeal fractures, shows similar imaging features and clinical characteristics across patients of different ages.

## Conclusion

The results of this study indicate that pediatric distal tibial S-H type II fractures demonstrate a high incidence of concomitant periosteal entrapment, with a predilection for the anterolateral region of the epiphysis. This phenomenon may occur even in fractures with initially minimal displacement. Periosteal entrapment is not a primary risk factor for fracture prognosis and therefore should not be considered an absolute indication for open reduction. Thus, even in the absence of MRI evaluation, if the initial closed reduction fails to achieve satisfactory articular alignment, a repeat closed reduction may be attempted. In summary, the primary treatment for such fractures should prioritize closed reduction to restore normal articular alignment; open reduction should be considered when closed reduction fails.

## Data Availability

The original contributions presented in the study are included in the article/Supplementary Material, further inquiries can be directed to the corresponding author.
